# Local or Not Local: Investigating the Nature of Striatal Theta Oscillations in Behaving Rats

**DOI:** 10.1523/ENEURO.0128-17.2017

**Published:** 2017-09-13

**Authors:** Laetitia Lalla, Pavel E. Rueda Orozco, Maria-Teresa Jurado-Parras, Andrea Brovelli, David Robbe

**Affiliations:** 1 Institut National de la Santé et de la Recherche Médicale, Unité 901, Marseille 13009, France and; 2 Aix-Marseille Université, Unité Mixte de Recherche S901, Marseille 13009, France; 3 Institut de Neurobiologie de la Méditerranée, Marseille 13009, France; 4 Institut de Neurosciences de la Timone, Unité Mixte de Recherche 7289, Aix-Marseille Université, Centre National de la Recherche Scientifique, Marseille 13385, France

**Keywords:** coherence, Granger, LFP, rat, striatum, theta oscillation

## Abstract

In the cortex and hippocampus, neuronal oscillations of different frequencies can be observed in local field potentials (LFPs). LFPs oscillations in the theta band (6–10 Hz) have also been observed in the dorsolateral striatum (DLS) of rodents, mostly during locomotion, and have been proposed to mediate behaviorally-relevant interactions between striatum and cortex (or between striatum and hippocampus). However, it is unclear if these theta oscillations are generated in the striatum. To address this issue, we recorded LFPs and spiking activity in the DLS of rats performing a running sequence on a motorized treadmill. We observed an increase in rhythmical activity of the LFP in the theta-band during run compared to rest periods. However, several observations suggest that these oscillations are mainly generated outside of the striatum. First, theta oscillations disappeared when LFPs were rereferenced against a striatal recording electrode and the imaginary coherence between LFPs recorded at different locations within the striatum was null. Second, 8% of the recorded neurons had their spiking activity phase-locked to the theta rhythm. Third, Granger causality analyses between LFPs simultaneously recorded in the cortex and the striatum revealed that the interdependence between these two signals in the theta range was mostly accounted for by a common external source. The most parsimonious interpretation of these results is that theta oscillations observed in striatal LFPs are largely contaminated by volume-conducted signals. We propose that striatal LFPs are not optimal proxies of network dynamics in the striatum and should be interpreted with caution.

## Visual Abstract

**Figure:**
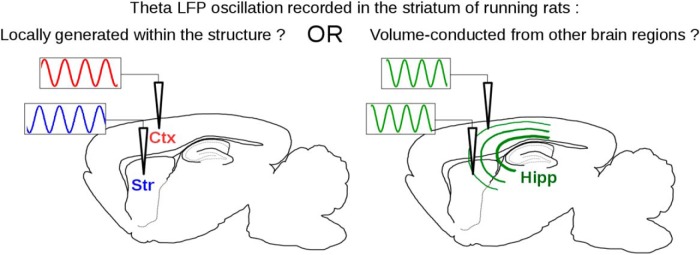

## Significance Statement

In the cortex and hippocampus, neuronal network oscillations can be observed in the local field potentials (LFPs) and contribute to information transfer between brain regions. LFP oscillations can also be recorded in the striatum, even if, unlike the cortex and hippocampus, this brain region's anatomic organization does not favor the generation of dipolar sources. It is therefore unclear if these striatal oscillations are locally generated or reflect volume-conducted signals generated distally from the striatum. Here, we provide evidence that striatal theta oscillations of the LFPs recorded while rats performed a running sequence are largely contaminated by volume-conducted signals. We propose that theta LFP oscillations in the striatum do not accurately reflect local cellular activity and should be interpreted with caution.

## Introduction

The striatum is remarkable in that it receives massive anatomic projections from the entire neocortex, but also from subcortical regions, such as the thalamus, hippocampus, and amygdala (Gerfen, 2004; Hintiryan et al., 2016; Hunnicutt et al., 2016). Moreover, the striatum processes behaviorally-relevant information from the somatosensory (Cho and West, 1997; Reig and Silberberg, 2014; Sippy et al., 2015; Kulik et al., 2017), motor (Koralek et al., 2012, 2013), and auditory cortices (Znamenskiy and Zador, 2013) and also from the hippocampus (van der Meer and Redish, 2011). Oscillations of the local field potentials (LFPs), which reflect mainly the coordinated synaptic activity of ensemble of neurons (Buzsáki et al., 2012), have been proposed as a mechanism supporting network-level computation (Buzsáki and Draguhn, 2004) and communication between brain areas (Fries, 2005; Bonnefond et al., 2017). In freely-moving rodents, LFPs recorded in the dorsal striatum display strong oscillatory power in the theta band (around 8 Hz) during behavioral tasks (Berke et al., 2004; Costa et al., 2006; DeCoteau et al., 2007a, b; Tort et al., 2008; Berke, 2009; Kimchi et al., 2009; Lemaire et al., 2012; Leventhal et al., 2012; Sturman and Moghaddam, 2012; Delcasso et al., 2014; Nakhnikian et al., 2014; Thorn and Graybiel, 2014; von Nicolai et al., 2014; Belić et al., 2016) and have been proposed as potential neural substrate for network-level computations relevant for locomotion and spatial navigation (DeCoteau et al., 2007a, b; Tort et al., 2008; von Nicolai et al., 2014).

Surprisingly, the neurophysiological mechanisms generating striatal theta oscillations are largely unknown. The striatum is composed at 95% by GABAergic medium spiny neurons, which are characterized by a spherical somatodendritic arborization and nonlayered cytoarchitectural organization (Gerfen, 2004). Thus, synaptic activity in the striatum is likely to generate closed-field configurations with small net contributions to the LFP (Johnston and Miao-Sin Wu, 1995; Berke, 2005; Boraud et al., 2005; Walters and Bergstrom, 2010; Einevoll et al., 2013). Moreover, due to the lack of recurrent excitatory input and the importance of local inhibition (Gerfen, 2004), the striatum has been proposed to be “not autonomously rhythmogenic” (Bracci, 2009). It is therefore unclear whether striatal theta LFP oscillations reflect the summation of local synaptic activities or volume-conduction effects from distant brain regions, such as the hippocampus (Buzsáki, 2002; Sirota et al., 2008).

To address this issue, we recorded spiking activity and LFPs in the dorsolateral striatum (DLS) of rats during the execution of a stereotyped running sequence. Subsequently, to disentangle potential inter-areal interactions from volume conduction effects, we performed functional connectivity analyses on LFP oscillations simultaneously recorded in the DLS and the forelimb primary somatosensory cortex (S1).

## Materials and Methods

All experimental procedures were conducted in accordance with standard ethical guidelines (European Communities Directive 86/60 - EEC) and were approved by the relevant national ethics committee (Ministère de l’enseignement supérieur et de la recherche, France, Ref 00172.01). 

### Animals

Long-Evans rats (*n* = 5, adult, males, 250–400 g) were housed in pairs (individually after surgery) in stable condition of temperature (22°C) and humidity (60%) with a constant light/dark cycle (12/12 h, all experimental procedures were performed during the light phase) and free access to food and water.

### Behavioral task

We used a DLS-dependent task for rats that favors the generation of a motor sequence with fine-tuned kinematic parameters (Rueda-Orozco and Robbe, 2015). In this task, rats are trained to run on a customized treadmill to obtain rewards according to a spatiotemporal rule. Once the treadmill was turned on, animals could stop it and receive a drop of sucrose solution by entering a “stop area” located at the front of the treadmill. In addition to this spatial rule, a temporal constraint was added: stopping of the treadmill was only effective if animals waited at least 7 s (goal time) before entering the stop area. If animals entered the stop area before the goal time, an error sound was played, and they were forced to run for 20 s. Initially, rats accelerated forward as soon as the treadmill was turned on and entered the stop area before the goal time, resulting in a majority of incorrect trials. After extensive training, rats executed a stereotyped sequence that could be divided in three overlapping phases: passive displacement from the front to the rear portion of the treadmill, stable running, and acceleration across the treadmill to enter the stop area. All rats were extensively trained to the task. The behavioral apparatus was controlled with custom-made LabView programs (National Instruments, RRID:SCR_014325).

### Surgery

Recording electrodes were chronically implanted under deep isoflurane anesthesia. For three rats (rats 001, 019, and 020), we used home-made tetrodes (nichrome wires, 12.5 µm in diameter, California Fine Wire, loaded on Neuralynx microdrives) targeting the right DLS (craniotomy centered at the following coordinates relative to bregma +0.6 mm AnteroPosterior (AP) and +0.35 mm MedioLateral (ML) and the depth −3.0 to −4.0 mm with respect to the surface of the brain). Tetrodes tips were gold plated to reduce their impedance to 100–200 kΩ at 1 kHz. For two rats (rats 027 and 032), we implanted two Buzsaki32 silicon probes loaded on Neuronexus microdrives targeting the DLS (AP = +1.2 mm, ML = +3.6 mm relative to bregma; depth = −3.0 mm relative to the surface of the brain) and the forelimb region of S1 (AP = −0.2 mm, ML = +3.8 mm relative to bregma, depth = −1.0 mm relative to brain surface, in order to target the layers Va). For all rats, a copper mesh protected the microdrive(s) and served as a local Faraday cage. Two miniature screws implanted above the cerebellum served as ground and reference. We confirmed the position of the electrodes with cresyl-violet staining after electrolytic lesions.

### Behavioral and neural data acquisition

Rats 001, 019, and 020 completed at least 65 sessions before the start of electrophysiological recordings. Rats 027 and 032 underwent surgery when naïve, and we considered for this study only data after the 45^th^ session. Neurophysiological signals were amplified 1000 times via a Plexon VLSI headstage and a PBX2 amplifier and acquired at 20 kHz on two synchronized National Instruments A/D cards (PCI 6254, 16-bit resolution). To determine the position of the animals, we used a CCD camera (scA640-70fc, Baser, 60 frames s^−1^, 9 pixels cm^−1^) positioned laterally to the treadmill and extracted the rat body's position with a custom-made program (LabView Vision, National Instruments, RRID:SCR_014325). Signal from the treadmill’s motor was recorded to identify precisely the beginning and the end of the trials. We only included in our analysis correct trials (longer than 7 s) during which animals perform the stereotypical “front-rear-front” running sequence. We worked on the following dataset for the analysis of LFPs:

**Table:** 

	Rat001	Rat019	Rat020	Rat027	Rat032
Number of sessions	11	12	11	13	14
Average number of trials per session ± SD	24 ± 6	29 ± 7	33 ± 10	64 ± 22	55 ± 22

### Preprocessing

Data analysis was performed using custom-made Matlab programs (RRID:SCR_001622) and the FieldTrip toolbox (http://www.fieldtriptoolbox.org/, RRID:SCR_004849). First, the data were down-sampled to 1250 Hz. Faulty channels were discarded on visual inspection with NeuroScope (http://neurosuite.sourceforge.net, RRID:SCR_002455). Artifact rejection was completed using FieldTrip visual rejection function: for each epoch and channel, the LFP signal variance and z-value were computed over time and inspected to detect artifact. The remaining data were bandpass filtered from 0.1 to 250 Hz, and a notch filter was added to remove electrical noise artifacts (integer multiples of 50 Hz). We divided the continuous signal into two types of epochs. “Run” epochs included the last 5 s preceding the arrival of the rat in the stop area. During these 5 s, the rats were continuously running. The “baseline” epochs included 5 s of intertrial time before the start of the considered trial.

### Spike-LFP entrainment

Spike sorting was performed semi-automatically using the clustering software KlustaKwik (http://klusta.readthedocs.io, RRID:SCR_014480) and the graphical spike sorting applications Klusters (http://neurosuite.sourceforge.net, RRID:SCR_008020) or KlustaViewa (http://klusta.readthedocs.io). Spike-LFP coupling was examined by producing phase histograms using FieldTrip function ft_spiketriggeredspectrum. Units yielding <20 spikes in total (across all the considered epochs) were excluded from the analysis. All units were visually inspected and, if several clusters corresponded to the same cell, supernumerary clusters were removed. To calculate the significance of spike-LFP entrainment, two metrics were computed: the Rayleigh *p* value (testing the nonuniformity of the circular distribution) and the pairwise phase consistency (PPC) values (Vinck et al., 2010). We found good accordance between these two methods in most cases, although on some instances (for low-frequency modulated cells; see Results) the Rayleigh *p* value was not specific enough of the frequency of entrainment. Hence, we considered cells as being specifically entrained to the theta rhythm if they also presented a maximum PPC at 8 Hz. To quantify the strength of the entrainment and the preferred phase for each unit, we hypothesized that the spike-phase distribution followed a von Mises distribution and we computed the concentration factor kappa κ and the preferred phase θ (Benchenane et al., 2010) using the Matlab Circular Statistics Toolbox (Berens, 2009). We worked on the following data set for the analysis of spike-LFP entrainment:

**Table:** 

	Rat001(11 sessions)	Rat019(12 sessions)	Rat020(7 sessions)	Rat027(6 sessions)	Rat032(7 sessions)	All rats
Number of units	36	159	143	124	35	
Number of units removed (less than 20 spikes)	−2	−20	−7	−5	−6	
Duplication correction	−9	−16	−16	0	0	
Total	25	123	120	119	29	416

### LFP spectral analysis

**Figure 1.**
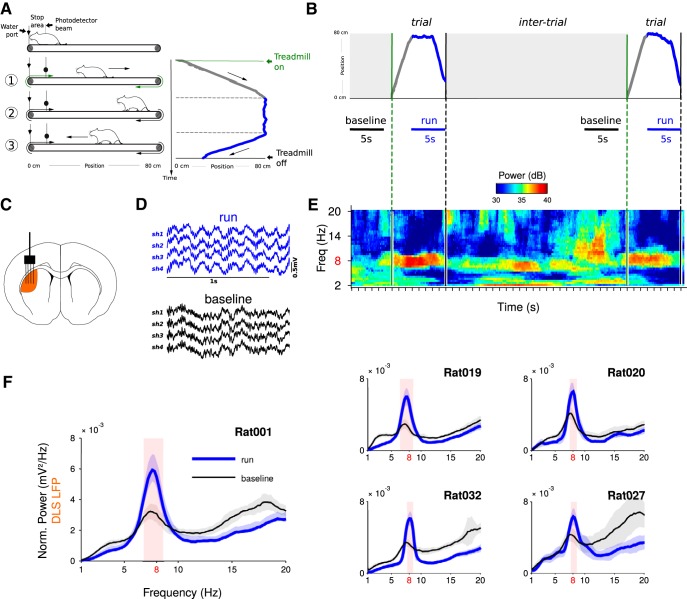
LFP oscillations recorded in the DLS are modulated by the execution of a stereotyped motor sequence. ***A***, Illustration of the front-rear-front running sequence performed by rats on a motorized treadmill (left) and the trajectory of an animal during a single trial (right). ***B***, Run and baseline are 5-s-long epochs chosen during trials and intertrials, respectively. ***C***, Schematic representation of electrodes position. ***D***, Raw LFP traces recorded with a four-shank silicon probe (a single channel per shank is shown). ***E***, Time-frequency power spectrogram during consecutive trials and intertrials. ***F***, Striatal LFP power spectra during run and baseline epochs (mean ± SD, average over electrodes and sessions). Power was normalized by 1/frequency^2^. Shaded red area indicates the frequencies at which the power is significantly different in run compared to baseline.

**Figure 2.**
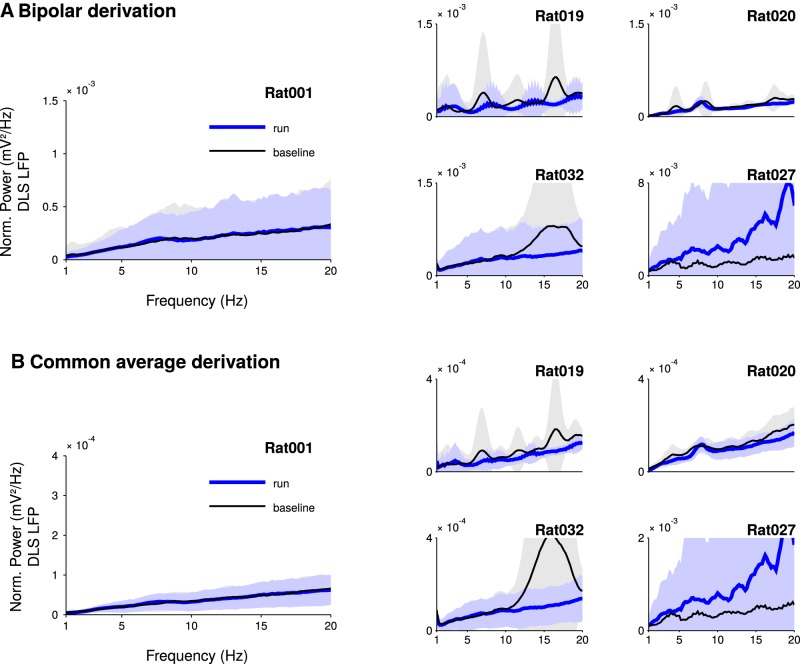
The prominent peak at theta frequency disappears from power spectra after LFPs rereferencing. ***A***, Striatal LFP power spectra during run and baseline using a neighboring shank or tetrode as reference (mean ± SD, average over electrodes and sessions). ***B***, Striatal LFP power spectra during run and baseline using the common average reference derivation (mean ± SD, average over electrodes and sessions). For ***A***, ***B***, the power was normalized by 1/frequency^2^. The power in the theta-band is not significantly different in run compared to baseline.

Power spectra were computed from 5-s epochs using a multitaper method based on discrete prolate spheroidal slepian sequences (Mitra and Pesaran, 1999) using the FieldTrip toolbox. For the time-frequency representation in Figure 1, spectral density was estimated for f = 2–20 Hz (in steps of 0.5 Hz) with nine orthogonal tapers and a spectral smoothing of 0.2 * f (parameters were chosen for graphical visibility). The logarithm was taken to present data in decibel. For the power spectra in Figure 2, spectral density was estimated for f = 1–20 Hz (in steps of 0.2 Hz) with seven orthogonal tapers and a spectral smoothing of 0.8 * f (parameters were chosen to characterize at best the 8 Hz frequency). Power estimates were computed across all trials within a recording session. For better visualization, the power at each frequency was multiplied by the frequency squared (“whitening”). To assess whether theta oscillations were locally generated, a bipolar derivation of the signals was performed using as reference the average LFP signal from another shank on the same silicon probe (∼200 µm away) or another tetrode on the same construct (∼350 µm away), instead of the animal external ground. This procedure relies on the assumption that volume conducted signal is equally present on all the channels and hence will be subtracted (Bastos and Schoffelen, 2016). A common average reference derivation was also performed by removing the average signal computed across all shanks or tetrodes.

### LFP coherence analysis

Spectral coherency was computed between striatal and cortical LFPs for frequencies f = 1–20 Hz (in steps of 0.2 Hz) with seven orthogonal tapers and a spectral smoothing of 0.8 * f (same parameters than for power estimation). Coherency is the cross-spectrum of striatal and cortical LFPs normalized by the square root of their respective power and is complex-valued. The modulus of coherency is the coherence, it is real valued between 0 and 1, and it measures consistency in phase difference between neural signals. If two signals are contaminated by a third (unique) common source, this would lead to coherence values at zero phase-lag, due to instantaneous field-spread. Thus, to eliminate potential confounds due to volume conduction, we also computed the imaginary part of the coherency, called imaginary coherence, which measures the degree of synchronization at non-zero time-lags (Nolte et al., 2004; Bastos and Schoffelen, 2016). Since the imaginary coherence is the projection of the complex coherency on the imaginary axis, values may be positive or negative. The coherence angle is the argument of the complex coherency. It reflects the phase-lag between two signals. A synchronization at zero phase-lag, resulting from passive field spread, will yield a coherence angle equal to zero and a null imaginary coherence.

### LFP Granger causality

To study the coupling between striatal and cortical signals, we used Granger causality analysis. Granger causality is a directed functional connectivity measure and reflects the degree of statistical predictability of one time series on another (Granger, 1980; Brovelli et al., 2004; Ding et al., 2006; Bressler and Seth, 2011; Seth et al., 2015). We used a nonparametric version of Granger causality, which allows the estimation from Fourier and wavelet transforms of time series data (Dhamala et al., 2008a, b). In addition, we exploited the notion that measures of Granger causality appear as a decomposition of total interdependence between two time series. In the time domain and for finite time series, this decomposition is expressed as follows:

$$F_{x,y}\;(t)=F_{x\to y}\;(t)+F_{y\to x}\;(t)+F_{x.y}\;(t)$$

and represents the total interdependence between X(t) and Y(t) (Geweke, 1982; Chicharro and Ledberg, 2012). F_x,y_ quantifies the dynamic increase of the total interdependence between two time series at a given point in time, in contrast to the static interdependence quantified by linear correlation. Such total interdependence is the sum of three Granger causality measures: two directed measures F_x→y_ and F_y→x_ plus the “instantaneous” Granger causality term F_x.y_, accounting for unconsidered common influences to the processes. The same formulation is valid in the frequency domain (Ding et al., 2006) at frequency ω,

$$f_{x,y}\;(\omega )=f_{x\to y}\;(\omega )+f_{y\to x}\;(\omega )+f_{x.y}\;(\omega )$$

Spectral Granger causality measures were computed between DLS and S1 LFPs, using FieldTrip function ft_connectivityanalysis, for frequencies f = 1–20 Hz (in steps of 0.2 Hz) with seven orthogonal tapers and a spectral smoothing of 0.8 * f (same parameters than for coherence estimation). The computation was done across all trials in one session, then averaged across sessions.

### Statistical analysis

Since power, coherence and Granger causality values are not normally distributed, we used a nonparametric test to assess if our data were significantly different between run and baseline epochs. We used a one-sided paired Wilcoxon test, corrected for the number of frequencies tested using a false discovery rate (FDR) approach (Genovese et al., 2002; Table 1).

**Table 1.** Statistical table

	Data structure	Type of test	*n*	Frequency significant if
a	Fig. 1, LFP power (striatum): non-normal distribution	One sided, paired signed-rank Wilcoxon test, corrected with FDR q = 0.05 (run vs baseline)	Nb of sessions:Rat001, *n* = 11Rat019, *n* = 12Rat020, *n* = 11Rat027, *n* = 13Rat032, *n* = 14	*p* < 0.001:Rat001, *p* < 0.0005Rat019, *p* < 0.0007Rat020, *p* < 0.0005Rat032, *p* < 0.0004Rat027, *p* < 0.0001
b	Fig. 3A,B, phase histogram: non-normal distribution	Circular Rayleigh test (Matlab circular statistics toolbox)	*n* > 20 spikes (Berke et al., 2004)	*p* < 0.01 (Berke et al., 2004)
c	Fig. 3C, preferred phase histogram: non-normal distribution	Circular Rayleigh test (Matlab circular statistics toolbox)	*n* = 35 specifically theta-modulated cells	*p* = 0.0158
d	Fig. 4, LFP power (cortex): non-normal distribution	One sided, paired signed-rank Wilcoxon test, corrected with FDR q = 0.05 (run vs baseline)	Rat027, *n* = 13 sessionsRat032, *n* = 14 sessions	*p* < 0.001:Rat027, *p* < 0.00012Rat032, *p* < 0.00012
e	Distribution of theta peak frequencies: non-normal distribution	Paired signed-rank Wilcoxon test (striatum vs cortex)	Rat027, *n* = 13 sessionsRat032, *n* = 14 sessions	*p* > 0.3:Rat027, *p* = 0.375Rat032, *p* = 0.625
f	Fig. 4, coherence: non-normal distribution	One sided, paired signed-rank Wilcoxon test, corrected with FDR q = 0.05 (run vs baseline)	Rat027, *n* = 13 sessionsRat032, *n* = 14 sessions	*p* < 0.001:Rat027, *p* < 0.0001Rat032, *p* < 0.0008
g	Fig. 4, imaginary coherence: non-normal distribution	One sided, paired signed-rank Wilcoxon test, corrected with FDR q = 0.05 (run vs baseline) (baseline vs 0) (run vs 0)	Rat027, *n* = 13 sessionsRat032, *n* = 14 sessions	Baseline vs 0:Rat027, *p* < 0.005Rat032, *p* < 0.005Run vs 0:Rat027, *p* < 0.004Rat032, *p* < 0.005Run vs baseline:Rat027, *p* < 0.0015Rat032, *p* < 0.0006
h	Fig. 6, Granger causality measures: non-normal distribution	One sided, paired signed-rank Wilcoxon test, corrected with FDR q = 0.05 (run vs baseline)	Rat027, *n* = 13 sessionsRat032, *n* = 14 sessions	Total interdependence:*p* < 0.00006Granger causality:*p* < 0.00018

## Results

Animals were trained to perform a stereotyped running sequence on a motorized treadmill. The sequence was composed of three phases: passive displacement from the front to the rear portion of the treadmill, stable running around the rear portion of the treadmill and acceleration across the treadmill to enter a reward area (Fig. 1*A*). Recording sessions consisted of several trials interleaved with intertrial periods during which the treadmill was turned off (Fig. 1*B*). Visual inspection of the recordings revealed that LFPs in the DLS (Fig. 1*C*) were very similar across electrodes and displayed theta oscillations during the running phase of the trials (Fig. 1*D*). This later observation was confirmed by means of time-frequency analyses of the LFPs (Fig. 1*E*).

Theta oscillations centered around 8 Hz were present during the run phase of the task, and showed a marked decrease in power during the intertrial period (Fig. 1*E*). To characterize the robustness of this task-dependent modulation, we systematically compared the oscillatory content of the LFPs between run epochs and a baseline taken during the intertrial, when the treadmill was off and animals were not running (Fig. 1*B*; see Materials and Methods). For all rats, power spectra showed a prominent peak around 8 Hz, significantly larger during run epochs compared to baseline (Fig. 1*F*; *p* ^a^ < 0.001 in all rats). This result is congruent with previous reports on the presence of theta oscillations in striatal LFPs in freely-moving rodents (Berke et al., 2004; Costa et al., 2006; DeCoteau et al., 2007a, b; Tort et al., 2008; Berke, 2009; Kimchi et al., 2009; Lemaire et al., 2012; Leventhal et al., 2012; Sturman and Moghaddam, 2012; Delcasso et al., 2014; Nakhnikian et al., 2014; Thorn and Graybiel, 2014; von Nicolai et al., 2014; Belić et al., 2016).

To assess whether striatal theta oscillations were locally generated rather than contaminated by volume-conducted fields generated outside the striatum, we conducted several analyses. First, we examined if theta oscillations were present after bipolar derivation between adjacent shanks (or tetrodes) or removal of common-average activity. Bipolar and common-average derivations allow the removal of common signals that may be due to sources external to the neural volume spanned by the recording electrodes. The results showed that the theta peak disappeared from the power spectra both for bipolar (Fig. 2*A*) and common-average reference derivations (Fig. 2*B*).

**Figure 3.**
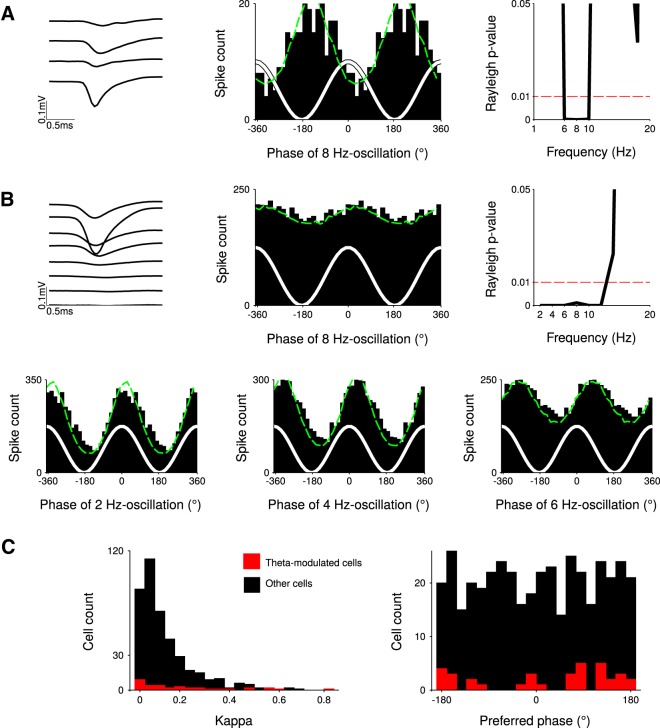
A minority of recorded striatal neurons is specifically entrained to theta oscillations. ***A***, Example of neuron whose firing pattern is specifically modulated by theta oscillations (6–10 Hz): waveforms (left), 8 Hz-phase histogram (middle, κ = 0.48, θ = −155°), and Rayleigh *p* value for each frequency between 1 and 20 Hz (right). ***B***, Example of neuron entrained by low frequency oscillations (1–12 Hz). Top, same as ***A***. Bottom, Additional phase histograms show a strong modulation at 2 Hz (κ = 0.58, θ = 9.2°) and 4 Hz (κ = 0.49, θ = 28°) but a weaker modulation at 6 Hz (κ = 0.24, θ = 69°) and 8 Hz (κ = 0.08, θ = 23°). ***C***, Population histograms of kappa and preferred phase, for theta-modulated cells (red) and other cells (nonmodulated cells and non-theta-modulated cells, black).

Second, to assess the local effect of striatal theta oscillations on single-neuron activity, we investigated if striatal neurons were entrained by the theta oscillations, i.e., if spikes occurred more often at certain phases of the theta oscillation. We constructed spike-phase histograms and tested for nonuniformity of the phase distributions using the Raleigh test. We analyzed the activity of 416 well-isolated neurons. A total of 8% (35/416) of the neurons displayed specific phase modulation at the theta frequency (Rayleigh *p* ^b^ < 0.01; Fig. 3*A*). Additionally, 14% (59/416) showed strong modulation by a slow component of the LFP with a concomitant weak entrainment at theta frequency (Fig. 3*B*). These neurons were not considered as being specifically theta entrained. The rest of the neurons did not display any significant modulation in the theta frequency range. To quantify theta phase modulation at the population level, we fitted, for each neuron, the distribution of its spike phases with a von Mises distribution. We computed the concentration factor κ, which measures the strength of the modulation, and the preferred phase θ (Benchenane et al., 2010). Neurons that were theta-modulated displayed a wide range of κ values and the majority was weakly modulated (κ ≤ 0.2 for 22 out of 35 theta-modulated units; Fig. 3*C*, left). The histogram of phase preferences for theta-modulated neurons did not exhibit a clear phase preference (Fig. 3*C*, right) and a test for nonuniformity distribution did not reach the significance level classically used for this kind of analysis (circular Rayleigh test, *p* ^c^ > 0.01; Table 1). Altogether, these results show that theta oscillations of the LFP weakly entrained spiking activity in the DLS.

**Figure 4.**
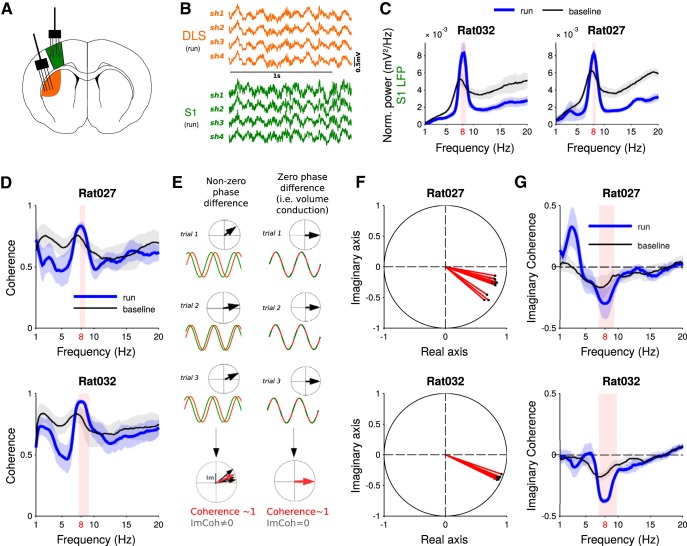
Coherence and imaginary coherence analyses. ***A***, Schematic drawing of the silicon probes position during simultaneous recordings in forelimb S1 and DLS. ***B***, Raw LFP traces recorded in DLS and S1 (a single channel per shank is shown). ***C***, Cortical LFP power spectra during run and baseline, for two rats. ***D***, Average coherence spectra during run and baseline, for two rats. ***E***, Schematic illustration of the two scenarios yielding a value of coherence close to 1: a stable phase coupling with non-zero phase difference and volume conduction. The imaginary part of the coherency, however, is different. ***F***, Complex coherency values at 8 Hz for all the sessions of the two rats during run (*n* = 13 and 12 sessions). ***G***, Averaged imaginary coherence during run and baseline. All graphs represent the average values across sessions ± SD. Shaded red area indicates the frequencies at which the power (***C***), the coherence (***D***) or the imaginary coherence (***G***) is significantly different in run compared to baseline.

Then, we tried to disentangle local contributions from volume conduction effects through the analysis of LFPs simultaneously recorded from the DLS and the forelimb primary somatosensory cortex (S1, 27 sessions in Rats 027 and 032; see Materials and Methods; Fig. 4*A*). Power spectra of the cortical LFPs were very similar to those of striatal LFPs (Fig. 4*B*) and presented a prominent peak around 8 Hz, significantly greater during run than baseline (Fig. 4*C*, *p* ^d^ < 0.001). For each session, the average peaks frequency in the theta range for striatal and for cortical LFPs were very similar (for Rat032: 7.80 ± 0.18 and 7.83 ± 0.19 Hz, respectively, and for Rat027: 7.80 ± 0.22 and 7.84 ± 0.12 Hz, respectively), and the two distributions were not different (Wilcoxon signed-rank test, *p* ^e^ > 0.3).

**Figure 5.**
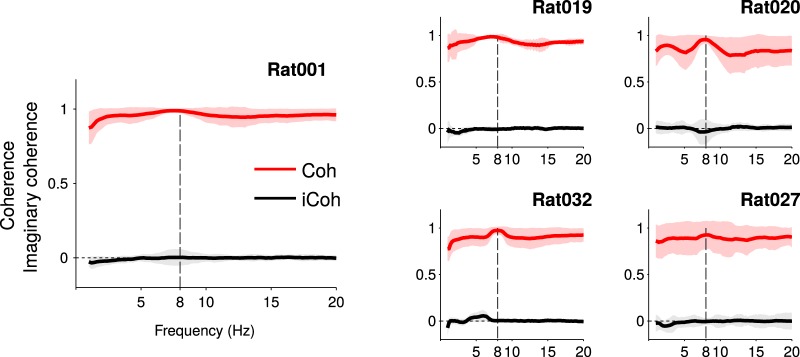
Coherence and imaginary coherence between different recording sites in the striatum, during run epochs, for all rats. Average values and SDs across all sessions are shown.

We then studied phase relations between theta oscillations of LFPs recorded simultaneously either in the cortex and the striatum or within these two brain regions. We first investigated phase relations between theta oscillations recorded in S1 and DLS (see Materials and Methods). Phase coherence between striatal and cortical LFPs in theta frequency range for both rats was significantly higher during run than baseline (Fig. 4*D*, *p* ^f^ < 0.001). However, it is known that coherence values can be biased by passive field spread: if oscillations generated in a distant brain area are volume conducted to both DLS and S1, they will reach both electrodes simultaneously, yielding high coherence values with zero phase-lag (Fig. 4*E*). Thus, we computed the imaginary coherence (i.e., the coherence at non-zero phase-lag; see Materials and Methods; Fig. 4*F*) between striatal and cortical LFPs. In both animals, we found that the imaginary coherence was different from zero during both run (*p* ^g^ < 0.004) and baseline epochs (*p* ^g^ < 0.005), with a magnitude significantly stronger during run than baseline (Fig. 4*G*, *p* ^g^ < 0.0015 and *p* ^g^ < 0.001). Second, we studied phase relations between LFPs recorded within the striatum. The average coherence between LFPs recorded at different sites within the DLS was very high for all rats (Fig. 5, Rat001: 0.99 ± 0.02; Rat019: 0.98 ± 0.02; Rat020: 0.96 ± 0.04; Rat 027: 0.96 ± 0.04; Rat032: 0.97 ± 0.04), but the imaginary coherence and coherence angle fell to zero values (imaginary coherence for Rat001: 0.00 ± 0.05; Rat019: −0.01 ± 0.02; Rat020: −0.04 ± 0.13; Rat027: 0.00 ± 0.02; Rat032: 0.00 ± 0.01; and coherence angle for Rat001: 0.00 ± 0.06; Rat019: −0.01 ± 0.02; Rat020: −0.04 ± 0.14; Rat027: 0.00 ± 0.02; Rat032: 0.00 ± 0.01), showing that LFPs recorded from different sites in the striatum are synchronized with zero-phase-lag. For Rat032, we also performed the same analysis on LFPs from different shanks of the silicon probe implanted in the cortex. Similarly, the average coherence between cortical LFPs was very high (0.98 ± 0.01 for Rat032) and average imaginary coherence and coherence angle fell close to zero values (for Rat032: 0.02 ± 0.02 and 0.02 ± 0.02, respectively).

**Figure 6.**
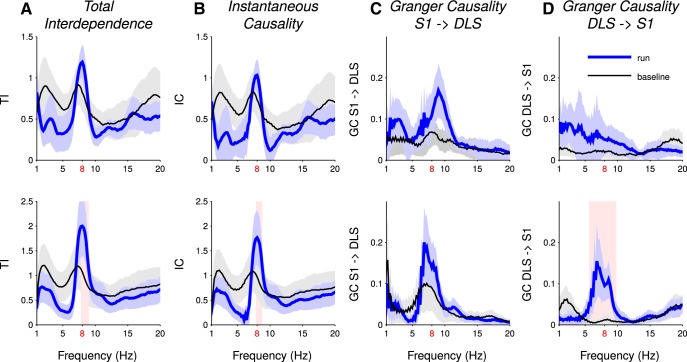
The total interdependence between DLS and S1 LFPs is mainly accounted for by instantaneous causality. ***A***, Total interdependence DLS/S1 during run and baseline, for Rat027 (top) and Rat032 (bottom). ***B***, Instantaneous causality during run and baseline, averaged across sessions, accounting for factors possibly exogenous to the (DLS, S1) system. ***C***, Directed Granger causality S1 → DLS during run and baseline. ***D***, Same as ***C*** for the direction DLS → S1. All graphs represent the average across sessions ± SD. Shaded red area indicates the frequencies at which the granger causality measures are significantly different in run compared to baseline.

Finally, we performed Granger causality analysis to further assess cortico-striatal directional influences and common influence from an external component. Granger causality analysis allows to estimate the total interdependence between two neural signals, defined as the sum of two directed measures of functional connectivity and an instantaneous measure representing the common influence of external factors on the two signals (see Materials and Methods). For both rats, the total interdependence exhibited a peak at 8 Hz during run epochs (Fig. 6*A*). Most (∼90%) of this total interdependence was accounted for by the instantaneous term (Fig. 6*B*). Directed causality measures contribute to the total interdependence to a much smaller extent, (∼10%; Fig. 6*C*,*D*). A significant increase in Granger causality from the DLS to the cortex was observed during the run phase in one animal (Fig. 6*D*, *p* ^h^ < 0.001).

## Discussion

Here, we recorded LFPs in the DLS of rats engaged in a motor task requiring to perform a fine-tuned running sequence. We compared the oscillatory content of the LFPs during running and resting periods and found a prominent increased rhythmical activity in the theta frequency band (6–10 Hz) during runs. Several observations nevertheless suggest that this theta rhythm is largely generated outside the striatum. First, the theta rhythm disappeared after rereferencing the LFPs to local neural activity and the imaginary coherence between LFPs recorded at different locations in the DLS was null. Second, theta oscillations of the LFP weakly entrained spiking activity in the DLS. Third, when LFPs were recorded simultaneously in the forelimb S1 and the striatum, Granger causality analysis revealed that the total interdependence between the two signals in the theta range was mostly accounted for by the instantaneous term, which reflects a potential common external source. Thus, striatal LFP oscillations in the theta frequency range appear to be largely volume-conducted signals and should not be interpreted as reflecting local network-level computation.

In the neocortex and hippocampus, LFPs reflect mainly the aggregate synaptic transmembrane currents occurring around extracellular recordings electrodes (Buzsáki et al., 2012) and oscillations of the LFPs have been shown to carry functionally-relevant network-level computation (Buzsáki, 2002; Buzsáki and Draguhn, 2004). In dorsal regions of striatum, LFP recordings have revealed rhythmical activity in various frequency bands, associated with specific behavioral or neuromodulatory states (Courtemanche et al., 2003; Masimore et al., 2005; Koralek et al., 2012, 2013). More specifically several studies have reported prominent rhythmical activity of the LFPs in the theta frequency range when rodents were engaged in locomotor activities (Berke et al., 2004; Costa et al., 2006; DeCoteau et al., 2007a, b; Tort et al., 2008; Berke, 2009; Thorn and Graybiel, 2014; von Nicolai et al., 2014), raising the possibility that theta oscillations reflect network level computation occurring in the striatum contributing to the processing of task-relevant information. Still, the spatial spread of electromagnetic fields may cause recording channels to pick up the activity of both local and distant neural sources. Indeed, results from literature reporting direct intracranial measurements indicate that LFPs can passively spread over several milimiters (Sirota et al., 2008) or even centimeters (Kajikawa and Schroeder, 2011) from their origins. This potential caveat may be exacerbated in a structure like the striatum whose anatomy favors the generation of closed field potentials (Johnston and Miao-Sin Wu, 1995; Gerfen, 2004; Walters and Bergstrom, 2010).

To the best of our knowledge, the strongest argument for a local source of theta oscillations in striatal LFPs is derived from a single study reporting that theta oscillations are still visible when the LFPs have been rereferenced against an intrastriatal electrode (DeCoteau et al., 2007a). However, local referencing can yield false positive results, depending on the exact position of the recording and references electrodes in regard of the oscillation source (Sirota et al., 2008). Indeed, subtracting two oscillatory signals with the same phase and frequency, but different amplitude (as it can happen due the passive attenuating effects of the brain tissue on LFPs) can result in an oscillatory signal with a preserved rhythmicity. Here, in agreement with previous report (Berke et al., 2004; Costa et al., 2006; DeCoteau et al., 2007a, 2007b; Tort et al., 2008; Berke, 2009; Lemaire et al., 2012; Leventhal et al., 2012; Sturman and Moghaddam, 2012; Delcasso et al., 2014; Nakhnikian et al., 2014; Thorn and Graybiel, 2014; von Nicolai et al., 2014; Belić et al., 2016), we found robust rhythmical activity of the LFPs during the running phase of our task, when the signal was referenced against an electrode placed above the cerebellum (Fig. 1). However, in contrast to what was observed in the aforementioned landmark study (DeCoteau et al., 2007a), this theta rhythmic activity totally disappeared when we rereferenced the signal against a striatal electrode or the common average signal of all striatal electrodes (Fig. 2). Unfortunately, there is no straightforward explanation for the discrepancy between our results and those obtained by DeCoteau et al. (2007a). Both works used tasks that are based on locomotion (rats running on a T-Maze or on a treadmill) and that massively engaged spiking activity in the DLS (Barnes et al., 2005; Rueda-Orozco and Robbe, 2015). In addition, the distance between electrodes was similar in both studies (from 200 to 600 µm). In our study, the lack of theta oscillation after local derivation was additionally supported by the fact that coherence was very high between LFPs recorded at different locations of the striatum while imaginary coherence and coherence angle were null. Thus, the similarity between the LFPs recorded in the striatum is most likely due to passive volume conduction. It could be argued that our results (imaginary coherence null between striatal recording sites and no theta oscillation in bipolar recording configuration) are compatible with theta oscillations being homogeneously generated throughout the entire striatum. This possibility is not well supported by the fact that 8% of the recorded striatal neurons were weakly entrained by theta oscillation (Fig. 3; Berke et al., 2004; DeCoteau et al., 2007a) with inconsistent phase preferences. Moreover, it has been shown that medium spiny neurons do not exhibit an autonomous rhythmic firing pattern (Mahon et al., 2006) nor resonance in the theta frequency range (Beatty et al., 2015).

The volume-conduction hypothesis is further substantiated by experiments in which we recorded LFPs simultaneously in the DLS and the forelimb somatosensory cortex. Like striatal LFPs, cortical LFPs displayed a prominent increase in rhythmical activity in the theta frequency band during runs. We performed Granger causality analyses that allows to dissect the interdependence between striatal and cortical LFPs into, on the one hand, directed measures of functional connectivity (that quantify interactions from striatum to cortex and vice versa) and, on the other hand, instantaneous causality values (that quantify the impact of common instantaneous influence on both striatal and cortical LFPs). Applied to our data, Granger analysis revealed that the total interdependence between theta oscillations of the LFPs recorded in the striatum and cortex is largely explained (at ∼90%) by a common external source driving both striatal and cortical theta oscillations of the LFPs. This raises the possibility that theta oscillations in the cortex were also contaminated by volume-conducted signals (Vinck et al., 2015a, b).

Altogether, our data strongly support the idea that theta oscillations observed in striatal LFPs are contaminated by volume-conducted signals. A good candidate for the origin of these signals is the hippocampus, as this structure generates prominent theta oscillations during running (Whishaw and Vanderwolf, 1973), which are known to passively spread over long distances (Sirota et al., 2008). In addition, the hippocampus sends excitatory projections to the ventral striatum (Gerfen, 2004) raising the possibility that theta oscillations are generated in this region and spread passively to more dorsal parts of the striatum. However, if this was the case, one could expect LFP theta power being stronger in the dorsal striatum than in the sensory cortex, which is not what we observed. Still we cannot exclude that the theta oscillations visible in the dorsolateral striatal LFPs result from the summation of fields generated in several brain regions (i.e., not only in the hippocampus). Determining precisely the nonstriatal origin of theta oscillations recorded in the DLS would require complex multi-sites electrophysiolgical recordings in behaving animals that fell beyond the scope of the present study.

Imaginary coherence analyses between striatal and cortical LFPs, quantifying the degree of synchronization at non-zero phase-lag, revealed significant non-zero imaginary coherence in the theta frequency band during run and rest. Since volume conduction is instantaneous, if theta oscillations were generated by a third-party source and passively spread to the striatum and cortex, one could have expected a null imaginary coherence between striatal and cortical theta. Thus, non-zero imaginary coherence could be interpreted as sign of functional coupling between both regions at theta frequency (von Nicolai et al., 2014). However, a non-zero imaginary coherence (i.e., a non-zero phase difference) between striatal and cortical theta LFPs may also arise from volume-conduction effects and phase-shifted theta generators located in adjacent brain regions. For instance, hippocampal theta oscillations are known to be generated by several sources that exhibit a phase shift (Sirota et al., 2008). This may, indeed, be responsible for the phase shift between striatal and cortical theta oscillations LFPs observed in our study. The fact that different hippocampal theta generators can be independently modulated during performance of a T-maze task (Montgomery et al., 2009) could also explain previously reported nonstationarites in the hippocampo-striatal coupling (DeCoteau et al., 2007b; Tort et al., 2008). Finally, non-null imaginary coherence in the theta band between cortex and striatum could also arise without functional coupling between these brain regions if a third-party theta source (e.g., the hippocampus) would synaptically modulate neuronal activity in the cortex and striatum with different delays (due to different connectivity schemes). In any case, our caution in interpreting the non-null imaginary coherence between cortical and striatal LFPs at theta frequency is reinforced by the small values of the directed measures obtained using Granger analysis (Fig. 6*C*,*D*; Nakhnikian et al., 2014; Belić et al., 2016), compared to the high value of instantaneous causality.

The contamination of striatal LFPs by volume-conducted theta oscillations is not incompatible with a subset of DLS neurons (8% in our study) being modulated at theta frequency (Berke et al., 2004; DeCoteau et al., 2007a). When considering all theta-modulated neurons, the strength of the modulation was generally weak and the different neurons did not exhibit a clear phase preference. These results support the idea that theta oscillations of the LFPs recorded in the striatum are largely volume conducted. The theta modulation of the firing rate could be due to either a direct influence from the medial entorhinal cortex, whose neurons exhibit theta-modulated spiking activity (Mizuseki et al., 2009) and project massively to the DLS (Kerr et al., 2007), or indirect projections from the hippocampus.

Our work, while not discarding the fact that a subset of striatal neurons have their activity coordinated at theta frequency, provides strong evidence for a prominent contamination of the striatal LFPs by theta oscillations generated distally. While our study focused on theta oscillations in the DLS, the interpretation concerns we raise is likely to apply to other frequency bands and subregions of the striatum. For instance, fast gamma oscillations of the LFPs recorded in the ventral striatum have been shown to be passively volume conducted from the piriform cortex, rather than locally generated (Berke, 2009; Carmichael et al., 2017). Interestingly, the spiking activity of ventral striatal neurons, which do not participate in the generation of the currents responsible for gamma LFPs oscillations was strongly gamma-modulated, most likely because the piriform cortex provides direct excitatory input to the ventral striatum. These results illustrate that, in a structure like the striatum, it is crucial to combine the study of spiking activity with complementary analysis methods to carefully assess the local origin of an LFP oscillation, even if it is strongly modulated at specific times of task performance. Finally, oscillations of the LFPs at theta frequency have also been recorded in midbrain regions, such as the ventral tegmental area (Harris Bozer et al., 2016) or the mesencephalic locomotor region (Noga et al., 2017). As in the striatum, the cytoarchitecture in these brain regions does not favor the generation of open fields and further investigation would be required to assess the locality of these oscillations. Altogether our work provides compelling support for recent publications that advised serious caution regarding the interpretation of LFPs (Buzsáki and Schomburg, 2015; Bastos and Schoffelen, 2016; Herreras, 2016).

## Synthesis

Reviewing Editor: Liset Menendez de la Prida, Instituto Cajal CSIC Decisions are customarily a result of the Reviewing Editor and the peer reviewers coming together and discussing their recommendations until a consensus is reached. When revisions are invited, a fact-based synthesis statement explaining their decision and outlining what is needed to prepare a revision will be listed below. The following reviewer(s) agreed to reveal their identity: Karim Benchenane, Antonio Fernandez-Ruiz

Your ms was independently evaluated by two reviewers and myself. We all agree it contains interesting data generating controversy in the field regarding the origin of striatal theta oscillations Thus we are all in favour of publication. However, for a fair debate we feel you may still need to address/discuss some issues more carefully.

- First, different connectivity between the dorsal and ventral striatum with hippocampal and entorhinal regions should be considered; as well as a role of vestibular signals (potentially engaging different circuits). See here my points and those by R1. Here, if you could add laminar recording data along the dorsoventral axis that would help. If not, please, discuss this point further.

- Second, previous bipolar recordings from other labs support some local signal (see comments by R1). You should consider whether your bipolar arrangement fully discard a local contribution.

- Third, both reviewers agree to suggest a more thorough population analysis of unit theta modulation

Regarding R1 comments on 1/f, I feel some normalization is desired, but chosing an appropiate band it not that straitforward. So I would not fully support recalculating the entire dataset. Instead, feel free to provide some complementary analysis to address this point.

I copy below reports from reviewers and my own indepedent assessment of your ms. Hope all this is helpful. Looking forward to read a revised version of this interesting paper.

----------------------

Report by R1:

In this study, the authors investigate the origin of theta oscillations in the striatum. The authors performed careful analyses showing that most of the theta oscillations comes from volume conduction, likely from the hippocampus.

In the second part, they provide more elaborate analyses showing some part of the theta recorded in the striatum could be still du to network activity in the S1-striatum network. Yet, even in this case, I believe that it does not convincingly eliminate the possibility that the theta-related measures come from volume conduction.

This is a very important article because many researchers are not aware about these problems of volume conduction, especially in structure with non-layered cytoarchitectural organization. For instance, it has also been showed that beta and gamma oscillations in the ventral striatum originate in the piriform cortex. For all these reasons this study is extremely important for the field.

Major comments

- The first part of the paper shows that both bipolar derivation and common reference derivation totally remove theta oscillations in the striatum. It is worth mentioning that the local referencing could yield significant values even in the complete absence of a local signal. However, in the case studied by the authors theta oscillations are totally removed which strongly suggest that theta oscillations are volume conducted. In the second part on the contrary the authors used the imaginary coherence between S1 and the striatum to states that some of striatal theta oscillations are not entirely volume conducted but could arise from the S1. Yet, since LFP in the CA3 pyramidal layer is phase shifted (150{degree sign}) relative to CA1 pyramidal layer and because CA1 and CA3 have different spatial distances to many distant brain sites (Sirota et al., 2008), theta-band synchronization between different sites that occurs at a phase delay different from 0{degree sign} or 180{degree sign} may thus reflect true, yet distant interactions between CA1 and CA3 (Sirota et al., 2008). Based on gross anatomical information, one could imagine that S1 is more influenced by CA1 whereas the striatum would be influence by a combination of CA1 and CA3 explaining a non-zeros phase lag between S1 and striatum. This problem is thus not solvable by constructing volume-conduction insensitive measures; it can only be addressed by using source reconstruction or current source density analysis which unfortunately is impossible in the striatum.

To conclude, the imaginary coherence (based on non-zero phase lag) could persist even in case of volume conduction based on multiple theta generators. Compared to these results, the bipolar derivation and the common reference provide more solid evidence for volume conduction.

- Is there any difference of phase lags between the different shank of the buzsaki probes in the cortex versus striatum?

- How do you reconcile your observation with the one made by De Coteau in 2007 in Journal of Neurophysiology that showed a persistent theta with bipolar recordings (cf. figure 2). Could this difference be due to the different tasks used? Is there a difference regarding the involvement of the striatum in the resolution of the task?

- The authors should comment in the discussion the impact of vestibular information on the generation of theta. It is known that passive displacement induces a theta that is slightly different from theta induced by real movement. In the 5sec epochs chosen for the analyses, there are two phases that are different relative to the vestibular information and in term of optic flow (first phase with rat's movement but without real displacement and thus no vestibular information and the second phase with both movement and displacement). Is there any difference between these two phases in term of theta power?

- The authors should discuss the origin of theta. Is this necessarily the hippocampus? Some studies showed that head movement are observed during rats' running at theta frequencies and head movement could be coherent with hippocampal theta oscillations. Is this still valid on the treadmill? Could this influence theta oscillations recorded in the striatum?

- The authors should provide a population analysis for theta modulation of striatal neurons (strength of modulation, and phase). Does the small number the theta modulated cells could be due to the fact that the DLS is not required in this task?

Minor comments

- Fig 1e please use an axis in the standard orientation

- The authors should avoid the normalization of the power spectrum by the 1/f component. It artificially removes the contribution to lower frequencies and over estimates the contribution of higher frequencies so that the respective prevalence of each frequency could be misleading. For instance, the authors say that 14% of the unit are modulated by slow component of the LFP that are not visible with the 1/f correction.

---------------------------------------

Report by R2

The present manuscript address an important question that is often disregarded, i.e. are the LFP oscillations recorded in a given structure (in this case the dorsal striatum) locally generated? The authors employ sound methods to provide convincing evidence that striatal theta oscillations are volume conducted from other structures.

The manuscript is clearly written and the conclusion of the authors accurately supported by the data provided, however several aspects can still be improved.

1- Unit analysis (Figure 3) provide interesting insights into DLS physiology, unfortunately they have been only briefly commented by the authors. One obvious conclusion does not seem to be clearly reflected in the Discussion, the fact that some cells are theta modulated indicates that despite the non-local nature of DLS theta oscillations, the structure does receive effective theta inputs. In addition to the two individual examples provided, Figure 3 should show population data. At least the theta phase distribution of spikes for all the modulated and non modulated cells separately. it would be also useful to plot the distribution of preferred theta phases for all cells. That will show if all the theta modulated cells tend to fire in the same phase.

2- Regarding the theta coherence of DLS and S1 there are several points that need clarification. The use of the imaginary part of the coherency to estimate non-zero lag synchrony is a valid methods although there are other perhaps more accurate in the present context, like the WPLI (weighed phase-lag index). More important, the existence of significant non-zero lag theta synchrony between DLS and S1 does not rule out that all recorded theta oscillations are volume conducted. Assuming that they are propagated from the HP, the fact that this is a big volumetric source that display a theta phase shift along it axis may account for this results, as both structures may sense with different intensity shifted theta fields. Additionally authors should quantify if there is any frequency difference between the theta recorded in both structures (paired comparison).

3- The manuscript will benefit from including a general discussion on how the present results can be generalized to other cases in which oscillations recorded with single-point electrodes are routinely assumed to be locally generated. This may be the case for theta recorded in areas close to limbic structures such as the HP or entorhinal cortex but also for different oscillations and brain regions. An important message from this paper is in my opinion the necessary caution in interpreting LFPs, specially in the absence of spatially resolved recordings.

----------------------------

Report by AE

Good paper generating controvery on the local nature of striatal theta activity. Well written and with carefull analysis supporting conclusions. Some alternatives should be considered though

- For a fair debate with existing literature authors should consider the fact that both in the Tort and De Coteau papers non-stationarities were reported for striatal-hippo coupling (and theta power) across the task suggesting that the degree of VC effect dynamically changes. Maybe VC sweeps across space depending on the dominant generators distributed along hippocampal layers and regions.

- Hippocampal and striatal regions are anatomincally connected. Specifically the subiculum and CA1 innervate preferentially the ventral striatum, which also receive from the entorhinal cortex. Authors should consider a more ventral location of striatal theta generators. Can authors provide some laminar recordings along the dorso-ventral axis?

- There is the possibility bipolar recordings fail to detect local components given the spatial organization of extracellular dipoles. For instance, striatal neurons morphology may poorly support spatial averaging to a local LFP signal. Authors should consider that 9% neurons reflected theta modulation, not fully discarding the possibility of some contribution. Actually, it is not the number of entrained cells but their targets what matters to build an LFP. Interneurons do not generate a field themselves, but their synaptic activity over allingned pyramidal cells.

## Author Response

We would like to thank the reviewers and editor for their thorough and constructive comments. We addressed them by performing additional analyses (addition of two panels in Figure 3, creation of a new Figure 5) and extensive rewriting of the discussion. Please find below a point-to-point reply to each comment. In some cases we generated figures and tables with the purpose of convincing the reviewers. In the text of our reply these figures and tables are referred to as Fig. R1 to R5 and Table R1 to R2). These figures and tables can be found at the bottom of our reply.

Reviewer #1

Major comments:

1.1 Based on gross anatomical information, one could imagine that S1 is more influenced by CA1 whereas the striatum would be influence by a combination of CA1 and CA3 explaining a non-zeros phase lag between S1 and striatum.

This problem is thus not solvable by constructing volume-conduction insensitive measures; it can only be addressed by using source reconstruction or current source density analysis which unfortunately is impossible in the striatum.

To conclude, the imaginary coherence (based on non-zero phase lag) could persist even in case of volume conduction based on multiple theta generators.

Reply to 1.1 We agree with the point made by Reviewer 1 (also raised by Reviewer 2). We realized that our manuscript was not addressing this issue clearly enough. We removed the sentence about the imaginary coherence in the abstract (page 1, line 15-16, striked out) and in the discussion (page 15, line 317-319, striked out), because the presence of non-null iCoherence does not exclude that theta oscillations are generated outside the striatum and the cortex (see below for a detailed explanation). We thought that this issue raised was worth emphasizing in the manuscript, and we added a detailed discussion paragraph (page 18, line 398-411) on the basis of the following arguments:

1) Results from literature (Sirota et al., 2008) suggest that hippocampal theta neural oscillations are not produced by a single dipolar source, but reflect the activity of several sources. Hippocampal theta oscillations can be recorded from distant cortical sites and the underlying sources may be phase-shifted.

2) The spatial spread of electromagnetic fields causes recording channels to pick up the activity of multiple neuronal sources. In our case, the electrodes were placed in the primary sensorimotor cortex (S1) and the dorsolateral striatum (DLS), and the recorded fields could have been produced by both local and distant neural sources. Indeed, results from literature reporting direct intracranial measurements indicate that passive spread of LFPs to sites can occur over distances more than a centimeter from their origins (Kajikawa and Schroeder, 2011). To conclude, the LFP recorded at S1 and DLS arise from the summation of multiple neural sources whose configuration (spatial extent, position and orientation) may vary across sources.

3) Given the previous two arguments and given that no source reconstruction could be performed due to the limited number of recording channels, we cannot infer neither the position nor the orientation of the potential underlying theta sources from current data. Thus, we cannot exclude that the non-zero imaginary coherence observed in our data could be produced by two distant coherent sources in CA1 and CA3 occurring with a phase lag different from zero, as hinted by the reviewer.

To conclude, we cannot exclude that a non-zero phase difference between striatal and cortical theta oscillations of the LFPs (as observed in our result of non-null iCoh) could be produced by interacting and phase-shifted theta sources outside the cortex and striatum.

1.2 Is there any difference of phase lags between the different shanks of the Buzsaki probes in the cortex versus striatum?

Reply yo 1.2 We performed an additional analysis to address this point. We calculated for each session, the coherence, the imaginary coherence and the coherence angle (reflecting the phase lag) between each shank located in the striatum and in the cortex during run epochs. (For Rat027, we could not calculate it for the cortex since 3 out of 4 shanks of the silicon probe implanted in the cortex became non-functional a few sessions after the start of the experiment). In brief the analysis confirmed the observation made with bipolar configuration.

For Rat032, in both regions, at 8 Hz, the coherence values were very high (average 0.97 {plus minus} 0.04 for the striatum and 0.98 {plus minus} 0.01 for the cortex). For the striatum, the imaginary coherence and the coherence angle were null (0.0036 {plus minus} 0.009 and 0.0037 {plus minus} 0.0095 respectively), showing no phase difference across all the shanks within the striatum. Similarly, for the cortex, the imaginary coherence and the coherence angle were close to zero values (0.0244 {plus minus} 0.0167 and 0.0249 {plus minus} 0.0173 respectively). However, a Wilcoxon rank-sum test between these two distributions (striatal angles vs cortical angles), revealed a significant difference between these distributions (p = 1.3857e-09). We cannot conclude that there is no difference of phase lags between striatal shanks and cortical shanks. For Rat027, the coherence between striatal shanks was very high (average across sessions = 0.96 {plus minus} 0.04) but the average imaginary coherence and the average coherence angle were null (-0.004 {plus minus} 0.02 and - 0.004 {plus minus} 0.02 respectively), showing no phase difference across all the shanks within the striatum. In this response letter, we provide the reviewer with a table (Table_R1) and a figure (Fig_R1) showing the coherence, the imaginary coherence and coherence angle between the different shanks of the striatum (similarly for the cortex) for an illustrative session of Rat032 and the average across all sessions, and for the different shanks of the striatum for Rat027.

We also performed this analysis for the other rats implanted with tetrodes in the DLS and found similar results. We now present these new results (zero-phase lag between theta in striatal LFPs) in a new figure (Fig. 5), in the text of the result section of the revised manuscript (page 13 line 281-291) and in the abstract (page 1 line 11-12). An additional explanation was also added in the Methods section (page 9, line 179-182). A sentence was added in the discussion explaining that this result confirms those obtained from the bipolar derivations (page 14 line 310).

Moreover, for Rat032, we computed the coherence and imaginary coherence between bipolar derivations in S1 and bipolar derivations in DLS. During run epochs, at 8 Hz, the average coherence is low (0.112 {plus minus} 0.05) and the average imaginary coherence is null (0.006 {plus minus} 0.09) showing a zero-phase lag between the two bipolar signals. We provide the reviewers a figure (Fig_R1_bis) showing the coherence and imaginary coherence between bipolar derivations in S1 and DLS. We did not add this result in the manuscript since it comes from a single animal.

1.3 How do you reconcile your observation with the one made by DeCoteau in 2007 in Journal of Neurophysiology that showed a persistent theta with bipolar recordings (cf. figure 2). Could this difference be due to the different tasks used? Is there a difference regarding the involvement of the striatum in the resolution of the task?

Reply yo 1.3 We agree with the reviewer that this an important point, even if we can only speculate on why DeCoteau et al. found persistent theta oscillations after bipolar derivation. We have now included a discussion paragraph in which we compared the differences and similarities between our behavioral task and the one of De Coteau et al. (page 16 line 354-359). First, it should be noted that both tasks are locomotion-based navigation tasks during which theta is most prominent when animal are running. Our task has been shown to require the DLS and strongly modulate striatal units (inactivation experiments and recordings have been performed and published, this information is now clearly stated page 4 line 78). The T-maze task used in De Coteau et al. also involve the DLS and previous work of Graybiel's team showed a strong modulation of striatal spiking activity (Jog et al.,1999; Barnes et al., 2005). Actually, there is no straightforward explanation for the discrepancy between our results and those obtained by DeCoteau et al. (2007a), since even the distance between electrodes were similar in both studies (from to 200 to 600 Âµm). We can only say that, as stated in Sirota et al., 2008: “we are dealing with multiple spatially segregated sources of various size and amplitude. Differential recordings simply measure the voltage difference and could yield significant values even in the absence of a local signal” (supplemental data, Sirota et al., 2008). Further work would be needed to better understand the reason for this discrepency.

1.4 The authors should comment in the discussion the impact of vestibular information on the generation of theta. It is known that passive displacement induces a theta that is slightly different from theta induced by real movement. In the 5sec epochs chosen for the analyses, there are two phases that are different relative to the vestibular information and in term of optic flow (first phase with rat's movement but without real displacement and thus no vestibular information and the second phase with both movement and displacement). Is there any difference between these two phases in term of theta power?

Reply to 1.4 We performed additional analyses and found a difference between these two behavioral phases in terms of theta power: an increased power when animals accelerate and cross the treadmill toward the reward area. A figure showing this effect is attached to this reply letter (Fig_R2). However, since rats increased their running speed while accelerating, we cannot disentangle running speed effects (Montgomery et al., 2009) from vestibular-specific contributions (we don't have the rats running at the same speed either while advancing on the treadmill or running without advancing). Hence, we cannot be conclusive on the impact of vestibular information.

1.5- The authors should discuss the origin of theta. Is this necessarily the hippocampus? Some studies showed that head movement are observed during rats' running at theta frequencies and head movement could be coherent with hippocampal theta oscillations. Is this still valid on the treadmill? Could this influence theta oscillations recorded in the striatum?

Reply to 1.5 The hippocampus is very well-known for the generation of a theta oscillation during locomotion, as added in the discussion of the revised manuscript (page 18, line 384-386). The previous result (described in Reply to 1.4) is interesting in that matter, as the running speed modulation of hippocampal theta power is well known. This shows that the recorded theta oscillation in the striatum has similar properties than hippocampal theta. Hence, one of the most parsimonious explanation is that we recorded the volume-conducted hippocampal theta. However, we did not provide any direct evidence confirming that the hippocampus is the source of the theta recorded in the striatum. Providing such evidence would require difficult experiments with multi-sites extracellular recordings (simultaneously in the hippocampus and the striatum) in behaving animals that fell beyond the primary goal of our work: determining if theta oscillations seen in the striatal LFP is locally generated. But to expend the discussion on the origin of theta oscillations, we also added a short section discussing the (unlikely) possibility that theta oscillations are generated in the ventral striatum (page 18, line 387-394, see also reply 3.2).

Regarding the head movements issue, it is indeed probable that rats display rhythmical head movements while running on the treadmill. However, head movements were not routinely recorded in all of our experiments. Nevertheless, we can assume the frequency of head movements to be different in the 3 phases of the task (passive displacement, running on the sport, acceleration). Still the frequency of the “striatal”theta was relatively stable in these 3 epochs (Fig 1e). Moreover, the peak frequency of head movements during the run epoch should be much greater than during the baseline epoch, which is not what we see in Fig 1f. Finally, the modulation of hippocampal theta by head movements has been shown to be weak (Ledberg & Robbe, PloS One, 2011). Hence, it is unlikely that the theta oscillations of striatal LFPs could be generated by sensorimotor input related to head movements.

1.6 The authors should provide a population analysis for theta modulation of striatal neurons (strength of modulation, and phase).

Reply to 1.6 This point was also raised by reviewer 2. We performed a population analysis for theta modulation of units, by fitting von Mises distributions to the spike-phase histograms and computing Kappa (strength of modulation) and theta (phase) parameters for each neuron. This analysis shows that neurons that were theta-modulated displayed a wide range of Îº values and the majority was weakly modulated (Îº {less than or equal to} 0.2 for 22 out of 35 theta-modulated units, Fig. 3c, left). The histogram of phase preferences for theta-modulated neurons did not exhibit a clear phase preference (Fig. 3c, right) and a test for non-uniformity distribution did not reach the significance level classically used for this kind of analysis (circular Rayleigh test, p c > 0.01, Table 1).

We now describe and discuss these results in the manuscript with two additional panels in figure 3, in the Results section on page 12, line 251-258, in the discussion (page 19, line 422-430) and in the Methods section (page 7, line 145-148). The lack of clear phase preference for the theta modulation of units and the weakness of the modulations is congruent with a source of oscillation external to the striatum.

1.7 Does the small number the theta modulated cells could be due to the fact that the DLS is not required in this task?

Reply to 1.7 Previously published inactivation experiments have shown that the DLS is required for the performance of this task with (Anonymous, in 20XX). Moreover this article showed that the spiking activity of striatal neurons are potently modulated by the task. The fact that the DLS is clearly involved in this task is now stated in the manuscript methods (page 4, line 78) and in the discussion section (page 16, line 355-356).

Minor comments:

1.8- Fig 1e please use an axis in the standard orientation

Reply to 1.8 Done

1.9 The authors should avoid the normalization of the power spectrum by the 1/f component. It artificially removes the contribution to lower frequencies and over estimates the contribution of higher frequencies so that the respective prevalence of each frequency could be misleading. For instance, the authors say that 14% of the unit are modulated by slow component of the LFP that are not visible with the 1/f correction

Reply to 1.9 This is a valid concern, as the choice of normalization often alter the visual rendition of the data. In this particular case, we chose to normalize by 1/fÂ² in order to compare more easily our date with a recent work by vonNicolai et al. (2014, Journal of Neuroscience). Nevertheless, we attached to this reply letter a representation of the power spectra for the 5 rats with different choices of normalization: either linear power, either in a logarithm scale or with the 1/fÂ² normalization. The peak in the theta frequency band is clearly prominent in the 3 forms of normalization (Fig_R3).

Reviewer #2

2.1 Unit analysis (Figure 3) provide interesting insights into DLS physiology, unfortunately they have been only briefly commented by the authors. One obvious conclusion does not seem to be clearly reflected in the Discussion, the fact that some cells are theta modulated indicates that despite the non-local nature of DLS theta oscillations, the structure does receive effective theta inputs.

In addition to the two individual examples provided, Figure 3 should show population data. At least the theta phase distribution of spikes for all the modulated and non modulated cells separately. it would be also useful to plot the distribution of preferred theta phases for all cells. That will show if all the theta modulated cells tend to fire in the same phase.

Reply to 2.1 We agree with the reviewer that we did not emphasize that some striatal cells are indeed theta-modulated. Indeed, we do observe that 8% of the recorded cells are specifically theta-modulated. Such spike-timing entrainment could be explained by a theta-modulated synaptic input. It has been shown by Kerr et al., (2007) that the medial entorhinal cortex sends strong projection to the dorsal striatum, thus providing a potential theta-modulated input. We added this comment in the discussion of our manuscript (page 19, line 427-430).

The reviewer suggestion about population analysis is entirely valid and it has also been raised by Reviewer 1. We refer the reviewer to our reply 1.6 to reviewer 1.

2.2 The use of the imaginary part of the coherency is a valid methods although there are other perhaps more accurate in the present context, like the WPLI (weighed phase-lag index).

Reply to 2.2 Indeed, other functional connectivity metrics would have be completely appropriate here. Actually, we also implemented the WPLI metric in preliminary analyses (with the Fieldtrip toolbox). We attach to this letter our WPLI results on some illustrative sessions and the average across all sessions for both rats (Fig_R4). The results are in agreement with those obtained using imaginary coherence. Since our main goal was to disentangle volume-conducted and locally-generated signals, based on our preliminary results, we found that the use of coherence and imaginary coherence was more easily understandable to explore this dimension. Because the WPLI results do not bring additional information and to keep our manuscript as clear as possible we did not include these results in the revised version of the manuscript.

2.3 More important, the existence of significant non-zero lag theta synchrony between DLS and S1 does not rule out that all recorded theta oscillations are volume conducted. Assuming that they are propagated from the HP, the fact that this is a big volumetric source that display a theta phase shift along it axis may account for this results, as both structures may sense with different intensity shifted theta fields.

Reply to 2.3 We completely agree with the point made by Reviewer 2 (also raised by Reviewer 1) and we realize that our manuscript was not clear enough regarding this issue. We refer the reviewer to our response to reviewer 1 (Reply to 1.1). In short, we cannot exclude that a non-zero phase difference between striatal and cortical theta oscillations of the LFPs (as observed in our data) could be produced by interacting and phase-shifted theta sources outside the cortex and striatum.

2.4 Additionally authors should quantify if there is any frequency difference between the theta recorded in both structures (paired comparison).

Reply to 2.4 We performed additional analyses to show that, within the same session, the peak frequency in the theta-range was the same in the cortex and the striatum (exactly the same or {plus minus} 0,2 Hz). This result can be found in Fig_R5 and the associated table Table_R2 (frequency peaks for all sessions, in the striatum and in the cortex). We performed a paired Wilcoxon signed-rank test on the distributions of the theta peak frequency in the striatum and in the cortex, for Rat032 (p = 0.625) and Rat027 (p = 0.375). In both rats, p > 0.05, so we cannot reject the null hypothesis that the two distributions are the same. This result is now added in the manuscript (page 13, line 265-268).

We attempted to perform this analysis on a trial by trial basis, but this was impossible to implement due to the variability of the power within a single epoch.

2.5 The manuscript will benefit from including a general discussion on how the present results can be generalized to other cases in which oscillations recorded with single-point electrodes are routinely assumed to be locally generated. This may be the case for theta recorded in areas close to limbic structures such as the HP or entorhinal cortex but also for different oscillations and brain regions. An important message from this paper is in my opinion the necessary caution in interpreting LFPs, specially in the absence of spatially resolved recordings.

Reply to 2.5 We agree with this comment. We have now included in the manuscript some extension of this issue regarding gamma oscillations in the ventral striatum, that have very recently been showed to be volume conducted (Carmichael et al, 2017) (page 20 Line 436-441). We also added a discussion (page 20 Line 444-447) about very recent publications studying theta oscillations in the VTA (Harris Bozer et al, 2016) and in the Mesencephalic Locomotor Region (Noga et al, 2017) in freely moving rodents, that lack a clear demonstration of the local generation of the theta oscillations (especially in experiments where animals are in locomotion and where the contamination by hippocampal theta is very likely). Finally, we included recent references regarding the spurious interpretation of LFPs in different frequency bands and different brain areas (page 20 Line 448-450).

Report by AE

3.1 For a fair debate with existing literature authors should consider the fact that both in the Tort and De Coteau papers non-stationarities were reported for striatal-hippo coupling (and theta power) across the task suggesting that the degree of VC effect dynamically changes.

Reply to 3.1 We agree with editor. We now discussed the fact that theta oscillations seen in the striatal LFPs could result from the superposition of fields generated by several phase-shifted theta sources in the hippocampus. These sources are known to be independently modulated during T-maze tasks (Montgomery et al, 2009) which could explain the non-stationarities reported by the Graybiel lab studies. We now present this argument in the discussion section of the manuscript (p19 line 411-413)

3.2 Hippocampal and striatal regions are anatomically connected. Specifically the subiculum and CA1 innervate preferentially the ventral striatum, which also receive from the entorhinal cortex. Authors should consider a more ventral location of striatal theta generators. Can authors provide some laminar recordings along the dorso-ventral axis?

Reply to 3.2 This is a good point and we now discuss it in the manuscript (p18 line 387-390). If the ventral striatum was the main source of volume-conducted theta oscillations in the DLS we would expect stronger theta power in the striatum than in the cortex which is the opposite to our results, as can be seen on Fig_R5: the theta power is always more important in the cortex than in the striatum. Moreover, a recent paper by Carmichael et al. (2017) investigated the origin of gamma oscillations in the ventral striatum and reported that their LFP data did not contain reliable epochs with delta, theta, or beta oscillations. In addition, in this paper, the spiking activity of striatal neurons was strongly gamma modulated but the authors showed that gamma oscillations were volume conducted from the piriform cortex. While it is likely that there is more theta modulation of spiking activity in the ventral striatum, due to the hippocampal excitatory afferents, it is not clear how this could generate open fields (due to spherical somatodendritic arborization of the principal cells and the non-layered cytoarchitectural organization of the striatum). Unfortunately we can not provide at the time laminar recordings along the DV axis of the striatum.

3.3 There is the possibility bipolar recordings fail to detect local components given the spatial organization of extracellular dipoles. For instance, striatal neurons morphology may poorly support spatial averaging to a local LFP signal. Authors should consider that 9% neurons reflected theta modulation, not fully discarding the possibility of some contribution. Actually, it is not the number of entrained cells but their targets what matters to build an LFP. Interneurons do not generate a field themselves, but their synaptic activity over aligned pyramidal cells.

Reply to 3.3 Indeed, some striatal cells have their spiking activity theta-modulated. As stated in our reply to Reviewer #2, this could be explained by the fact that the DLS receives theta-modulated inputs, probably from the medial entorhinal cortex (Kerr et al., 2007). It is also possible that these cells participate to the generation of a local theta oscillations within the striatum. Indeed, if all the modulated striatal cells were interneurons with the same phase preference, they could modulate many MSNs. However, we performed a population analysis on the theta-modulated cells (see Reply to 1.6 and new panel in Figure 3) and observed no clear theta phase preference for all theta-modulated cells. Moreover, MSNs are not aligned in the striatum and their radial somatodendritic arborization is prone to produce closed-fields.

Finally, if interneurons were modulating MSN at theta frequency, a majority of units should have their firing rate modulated at theta frequency, which is not what we observed. Of course it is still possible that these modulations are subthreshold, even if intracellular recordings of striatal neurons in awake animals have not revealed such subthreshold theta oscillations (Mahon et al., 2006). Finally, even if there is some local theta “hidden” below the volume-conducted theta this does not affect the main conclusion of our paper: the prominent theta oscillation of the LFPs recorded in the DLS is largely contaminated by volume conducted signals and should not be used as a proxy of local network activity.
